# Oxidative stress induced sperm DNA damage, a possible reason for male infertility

**Published:** 2015-09

**Authors:** Md Bayejid Hosen, Md Rakibul Islam, Firoza Begum, Yearul Kabir, M Zakir Hossain Howlader

**Affiliations:** 1 *Department of Biochemistry and Molecular Biology, University of Dhaka, Dhaka-1000, Bangladesh.*; 2 *Department of Obstetrics and Gynecology, Bangabandhu Sheikh Mujib Medical University (BSMMU), Dhaka-1000, Bangladesh.*

**Keywords:** *DNA damage*, *Lipid peroxidation*, *Male infertility*, *Oxidative stress*, *Sperm quality*

## Abstract

**Background:**

Sperm DNA damage is an important factor in the etiology of male infertility.

**Objective:**

The aim of the study was to evaluate the association of oxidative stress induced sperm DNA damage with the pathogenesis of male infertility.

**Materials and Methods:**

The study comprised a total of 66 subjects, including fertile men (n=25) and infertile men (n=41) matched by age. Seminal malondialdehyde (MDA), phospholipid hydroperoxide (PHP), superoxide dismutase (SOD), total antioxidant status (TAS) and 8-hydroxy-2'-deoxy guanosine (8-OHdG) were estimated by spectrophotometric and ELISA based methods and the association with the sperm parameters was assessed.

**Results:**

The percentages of motile and morphologically normal cells were significantly lower (p < 0.001, p <0.001, respectivly) in infertile men. Seminal levels of MDA, PHP and 8-OHdG were significantly higher (p < 0.001, p < 0.001, and p=0. 02, respectively) while the SOD and TAS were significantly lower (p=0. 0003, p< 0.001, respectively) in infertile men. Sperm parameters were negatively correlated with MDA, PHP and 8-OHdG while positively correlated with SOD and TAS. A positive correlation of 8-OHdG with MDA and PHP and a negative correlation with TAS and SOD were also found.

**Conclusion:**

These results suggested that oxidative stress induced sperm DNA damage might have a critical effect on the etiology of infertility. Therefore, evaluation of oxidative status, antioxidant defense systems and DNA damage, together with sperm parameters might be a useful tool for diagnosis and treatment of male infertility.

## Introduction

ءale infertility is a common problem arises when the man is unable to produce or deliver fully functioning sperm. From the medical point of view, a couple is considered infertile if pregnancy does not occur within one or two years of unprotected intercourse. About 10% to 15% of couples are suffering from infertility worldwide (1). In developing countries like Bangladesh, the male infertility is like a curse and a recent study showed that 60% men of infertile couples are directly or partially responsible for the infertility (2).

The etiology of male infertility is poorly understood, though the causes of infertility are anatomic defects, endocrinopathies, immunologic problems, gene mutation, radiation, chemotherapy, ejaculatory failures and environmental exposures (3). In many cases, the identification of the exact factor responsible for male infertility is quite difficult, and the mechanisms of the underlying defects of poor semen quality remain obscure. Standard semen analysis using a light microscope has been widely used in most laboratories for initial evaluation of male fertility potential (4); however, diagnosing defective sperm function by standard semen analysis is difficult because the spermatozoon is a highly specialized cell that expresses a diverse array of biological properties to achieve fertilization (5). In addition, results of standard semen analyses can be very subjective and prone to intra and inter-observer variability (6). An individual’s semen quality can vary widely due to factors such as days of abstinence from ejaculation, febrile illness, stress and even problems with sample collection.

Oxidative stress is an important factor which influences fertility potential and elevated seminal Reactive Oxygen Species (ROS) can cause sperm dysfunction through lipid peroxidation of the sperm membrane (7-9). Therefore, though small physiological levels of ROS are essential for normal sperm functions, i.e. sperm capacitation, the acrosome reaction and sperm-oocyte fusion (10, 11), oxidative stress has been shown to be an important cause of male infertility (7-9). However, production of excessive amounts of ROS in semen can overwhelm the antioxidant defense mechanisms of spermatozoa and seminal plasma that results oxidative stress.

Several studies suggested that ROS attacks the integrity of DNA in the sperms’ nucleus by causing nucleotide modifications, DNA strand breaks and chromatin cross-linking (12, 13). Since spermatozoa have limited defense mechanisms against oxidative free radical attack on their DNA, therefore, 8-hydroxy-2′-deoxyguanosine (8-OHdG) is produced during oxidative DNA damage. It was reported that sperm DNA damage is closely related to male infertility and 8-OHdG is a sensitive marker of oxidative DNA damage caused by ROS in human sperm (14).

The assessment of seminal oxidative stress and sperm DNA damage, together with sperm parameters may play an important role in the diagnosis and treatment of male infertility. In the current study, we investigated the possible association of oxidative stress induced sperm DNA damage with male infertility. Here, it was measured seminal MDA, PHP and SOD activity as markers of oxidative stress while TAS and SOD as the oxidative defense in the study subjects. As a biomarker of DNA damage, seminal 8-hydroxy-2'-deoxy-goanosine (8-OHdG) level was measured in semen plasma.

## Materials and methods

**Study population**

The study was a case control study and conducted on 66 subjects (41 infertile male, age 26-42 years and 25 healthy volunteers, who have the experience of being father, age 24-44 years) matched by age. According to the World Health Organization (15), men are considered fertile when their standard semen analysis showed over 15 million sperm per milliliter semen, 50% are motile, over 4% are in normal morphology with some other satisfactory factors. In this study, it was considered the healthy fertile subject who had no chronic clinical illness and had their baby within one year of unprotected sexual intercourse. Infertile male was recruited from Bangladesh Assisted Conception Centre (BACC) & Womens’ Hospital (Pvt.) Ltd. Dhaka, Bangladesh. All infertile males included in this study had a minimum of one year of regular unprotected intercourse with their respective female partner. The female partners of these men had no history of untreated female-factor infertility and had a normal reproductive and sexual history as well as normal investigation. 

All participants were given an explanation of the nature of the study and informed consent was obtained. This study was approved by the ethical committee of Bangladesh Medical Research Council under the guidelines of the Ministry of Health and Family Welfare.

**Sample collection**

Semen samples were obtained from December 2012 to May 2013. All the samples were collected in a sterile container by masturbation (without using any lubricant that can harm sperm cells), after a period of 48 to 72 hours of sexual abstinence. After liquefaction aliquots of semen were centrifuged and semen plasma was isolated and stored at -20 C for the assay of oxidative stress markers. The assay was completed by June 2013.

**Standard semen analysis**

Following liquefaction, semen samples were evaluated for semen volume, appearance, pH, and viscosity. Routine semen analysis was performed according to World Health Organization guidelines (15) to determine sperm concentration and motility. For mortility analysis, it was considered total motility. Sperm concentration was expressed as concentration, i.e. ×10^6^/ml semen, while motility and morphology were expressed as a percentage. For the assessment of sperm morphology the smears of the raw semen were stained using the Diff-Quik kit (Allegiance Healthcare Corporation, Inc., McGaw Park, IL, USA). Immediately after staining, the smears were rinsed in distilled water, air-dried, and scored. Morphology was expressed as percentages of normal cells (without any abnormalities, i.e. head, mid-piece or tail abnormality)

**Assay of oxidative stress markers**

In this study, it was measured the values of malondialdehyde (MDA), phospholipid hydroperoxide (PHP) and superoxide dismutase (SOD) as oxidative stress markers. It was also measured total antioxidant status (TAS) as the ability to protect from oxidative stress. The MDA value was measured following Yagi method (16). 100 µL of semen plasma was taken in a glass test tube and 900 µL of normal saline was added in the tube. Then the lipoprotein portion was precipitated by trichloroacetic acid. The solution was boiled with thiobarbituric acid, which reacted with MDA and formed a pink color. The absorbance of the color was measured at the wavelength of 535 nm. PHP value was determined by the method of Miyazawa (17) based on the oxidation of ferrous to ferric ion in the presence of xylenol orange. 100 µL of semen plasma was added in 1 mL of working reagent. After 15-20 minutes of incubation the color formed in this reaction was measured at 520 nm. The determined values for MDA and PHP were expressed as nmol/ml.

SOD value was examined by commercial reagent kit (Sigma-Aldrich, USA) (18). The kit contained xanthine oxidase enzyme that produced superoxide anion which reacted with Dojindo’s highly water-soluble tetrazolium salt, WST-1 {2-(4-Iodophenyl) -3-(4-nitrophenyl)-5-(2, 4-disulfophenyl)-2H-tetrazolium, monosodium salt} that produced a water-soluble formazan dye. SOD inhibits the formation of the dye, therefore SOD activity is inversely proportional to the dye. 20 µL of semen plasma was added into 200 µL of working reagent. Then 20 µL of enzyme solution was added. After 20 minutes of incubation at 37 ^°^C the absorbance of the dye was measured at 450 nm by ELISA reader and the SOD activity was expressed as percentage.

TAS was also measured by antioxidant assay kit (Sigma-Aldrich, USA) (19). Metmyoglobin and hydrogen peroxide produced feryl-myoglobin radical which oxidized the ABTS (2, 2'-azino-bis (3-ethylbenzthiazoline-6-sulfonic acid) to produce a radical cation, ABTS+, a soluble chromogen that is green in color. Antioxidants suppress the production of the radical cation and the color intensity decreases proportionally. 10 µL of semen plasma was added in 20 µL metmyoglobin working solutions. Then 150 µL of ABTS substrate working solution was added. After 5 minutes of incubation 100 µL of stopping solution was added. Finally, the color intensity was measured at 405 nm by ELISA reader and the value for TAS was expressed as mM.

**Assay of DNA Damage**

As a biomarker of oxidative DNA damage, 8-hydroxy-2'-deoxy-goanosine (8-OHdG) was measured in semen plasma. The value was assayed by 8-OHdG EIA kit (Cayman, USA) (20). 

This assay was based on the competition between 8-OHdG and an 8-OHdG-acetylcholinesterase (AChE) conjugate (8-OHdG tracer) for a limited amount of 8-OHdG monoclonal antibody. 8-OHdG Tracer and 8-OHdG bound monoclonal antibodies bound to goat polyclonal anti-mouse IgG that were previously attached to the plate well. The plate was washed to remove unbound reagents and then Ellman’s reagent (which contained the substrate to AChE) was added into the well. 50 µL of semen plasma was added to this reaction. The product of this enzymatic reaction had a distinct yellow color and the absorbance was measured at 412 nm by an ELISA reader. The amount of free 8-OHdG was inversely proportional to the color and the determined value for 8-OHdG was expressed as ng/ml.

**Statistical analysis**

All the results were expressed as mean±SEM. The statistical analysis of the data was carried out with statistical package of social sciences, version 17.0, SPSS Inc, Chicago, Illinois, USA and Graph pad Prism version-5. 

The comparisons between two groups were tested by independent samples t-test. A 95% confidence interval was used. P values less than 0.05 were considered as statistically significant. The correlation between two continuous outcomes among infertile men was evaluated using Pearson correlation coefficient.

## Results

Statistically significant differences among fertile and infertile men are indicated in table I and table II along with their significant values.

**Semen quality in the study subjects**

The sperm count in fertile men was significantly higher (p<0.001) than infertile men (Table I). The percentages of motile and morphologically normal cells were significantly higher (p<0.001) in fertile men compared with infertile men (Table I).

**Oxidative status and DNA damage in study subjects**

The seminal levels of malondialdehyde and phospholipid hydroperoxide were significantly higher (in both cases p<0.001) in infertile men than in fertile men (Table II). On the other hand, the seminal superoxide dismutase activity and levels of total antioxidant status were significantly higher (p<0.001) in fertile men than in infertile men (Table II). The seminal levels of 8-hydroxy-2'-deoxyguanosine in fertile men were significantly lower (p<0.001) than in infertile men (Table II).

**Correlation of different parameters among infertile men**

**Figure 1:**
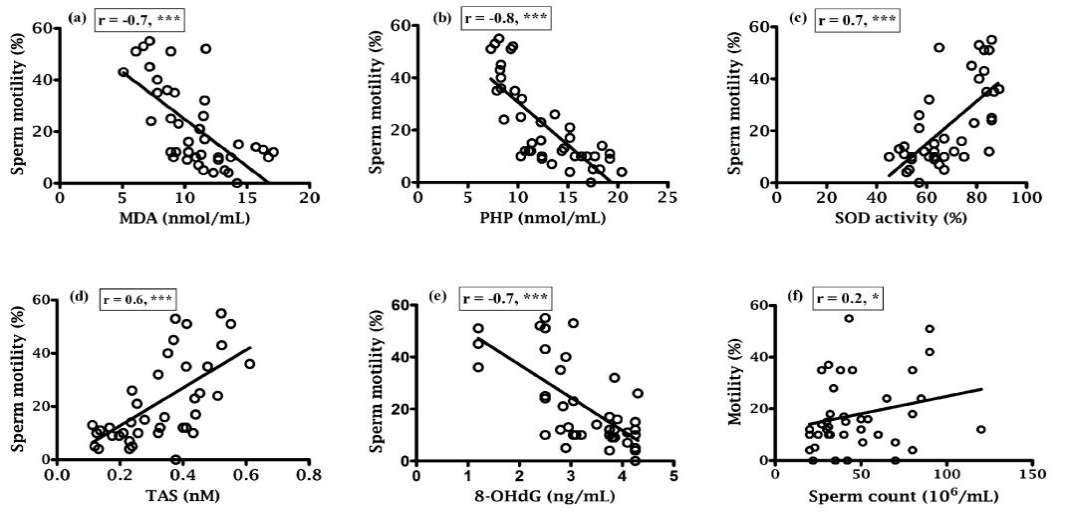
Correlation of sperm motility with (a) MDA, (b) PHP, (c) SOD, (d) TAS, (e) 8-OHdG and (f) Sperm count.

**Figure 2:**
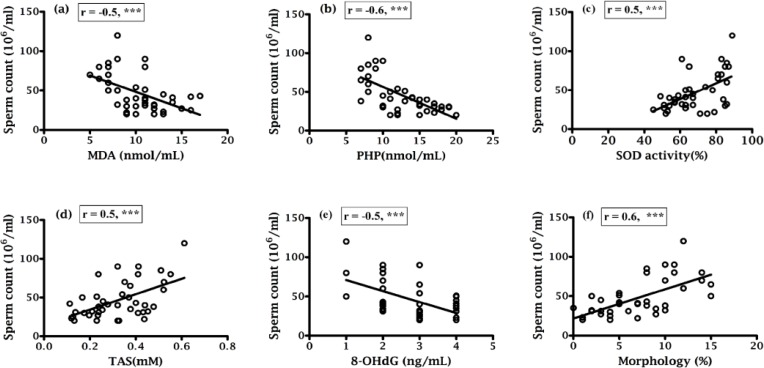
Correlation of sperm count with (a) MDA, (b) PHP, (c) SOD, (d) TAS, (e) 8-OHdG and (f) Morphology.

**Figure 3:**
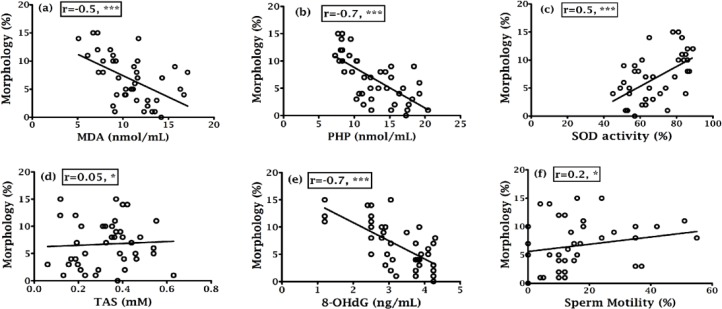
Correlation of sperm morphology with (a) MDA, (b) PHP, (c) SOD, (d) TAS, (e) 8- OHdG and (f) Morphology.

**Figure 4:**
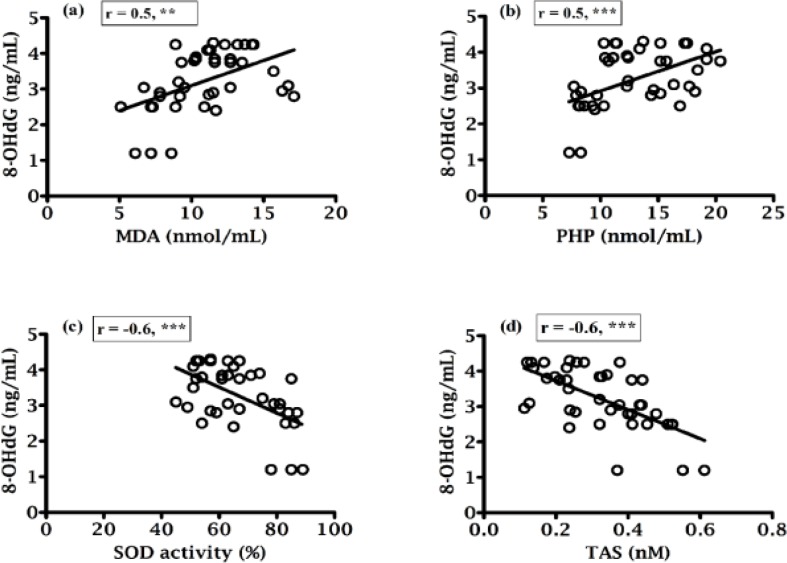
Correlation between 8-OHdG with (a) MDA, (b) PHP, (c) SOD and (d) TAS.**= p<0.0; (Significant). ***= p<0.001; (Highly significant).

Correlation coefficients of various parameters were indicated in figures 1, 2, 3 and 4 along with their significant values. According to figures 1a, 1b and 1e, there was a negative correlation of sperm motility with MDA, PHP and 8-OHdG while there was a positive correlation of sperm motility with SOD and TAS (Fig. 1c and 1d). On the other hand, although there was a positive correlation between sperm motility with sperm count (Fig. 1f), but that was not statistically significant, which indicate sperm motility does not depend on the sperm count. While there was a negative correlation found between sperm counts with MDA, PHP and 8-OHdG (Fig. 2a, 2b and 2e respectively), a positive correlation with SOD, TAS and morphology (Fig. 2c, 2d and 2f respectively) was existed. The morphology was negatively correlated with MDA, PHP and 8-OHdG (Fig. 3a, 3b and 3e respectively) while SOD was positively correlated and there was an insignificant positive correlation with TAS and motility (Fig. 3c, 3d and 3f respectively). In case of healthy control, correlation analysis of morphology with motility showed insignificant positive correlation. As shown in figure 4, there was the positive correlation of 8-OHdG with MDA and PHP indicating oxidative DNA damage while negative correlation with SOD and TAS (Figure. 4a to 4d, respectively).

**Table I:** Standard semen analysis in the fertile and infertile men

**Parameters**	**Healthy fertile men (n=25)**	**Infertile men (n=41)**	**p-value**
Sperm count (million/mL)	100.8 ± 3.84	46.63 ± 3.80	< 0.001
Motility (%)	61.56 ± 0.63	17.68 ± 2.17	< 0.001
Morphology (%)	39.92 ± 0.99	6.73 ± 0.66	< 0.001

Independent samples t-test was done as the test of significant. p<0.05 was taken as the level of significant.

Data were presented as mean ± SEM.

**Table II:** Different parameters in semen plasma of the fertile and infertile men

**Parameters**	**Healthy fertile men (n=25)**	**Infertile men (n=41)**	**p-value**
MDA (nmol/mL)	4.29 ± 0.21	10.88 ± 0.46	< 0.001
PHP (nmol/mL)	5.08 ± 0.21	12.80 ± 0.60	< 0.001
SOD activity (%)	91.04 ± 0.87	68.02 ± 2.06	0.0003
TAS (nM)	0.78 ± 0.03	0.33 ± 0.02	<0.001
8-OHdG (ng/mL)	1.7 ± 0.14	3.2 ± 0.13	0.02

Independent samples t-test was done as the test of significant. p<0.05 was taken as the level of significant, Data were presented as mean ± SEM.

MDA: Malondialdehyde

PHP: Phospholipid hydroperoxide

SOD: Superoxide dismutase

TAS: Total antioxidant status

8-OHdG: 8-hydroxy-2'-deoxy guanosine

***= p<0.001 (Highly significant).

***= p<0.001 (Highly significant).

***= p<0.001 (Highly significant).

## Discussion

Numerous factors have already been identified that can lead to male infertility; however, the underlying mechanisms that cause qualitative and/or quantitative defect of the spermatozoa are still unclear in many cases of male infertility. Several research groups investigated on male infertility and reported reductions in sperm count, sperm motility and morphology in infertile men (21, 22).

In the present study, it was found that sperm count and sperm motility were significantly (in both cases p<0.001) decreased in infertile men (Table I) that support other previous studies (23), which indicated that the sperm in infertile subjects were not only decreased in number, but the existing sperm were poor in motility. The sperm plasma membrane is particularly susceptible to lipid peroxidation by ROS due to the presence of high concentration of polyunsaturated fatty acids, which eventually lead to the loss of membrane fluidity and integrity; hence lose their competence for the membrane fusion events during fertilization. MDA is generally used as a marker of oxidative stress and in accordance with some studies (9, 23).Our study showed a higher degree of seminal oxidative stress in the form of MDA and PHP (in both cases p<0.001) in infertile men compared with fertile men. Other studies also reported elevated seminal MDA concentration in patients with oligozoospermic and azoospermic groups (24, 25).

SOD is one of the important elements of seminal plasma.Superoxide anion scavenging capacity and several investigators have reported reductions of SOD activity in semen of infertile men (26, 27). In this study, it was found seminal SOD activity and TAS levels in infertile men were significantly lower (p=0.0003, and p<0.001, respectively) than in healthy fertile men (Table II). Murawski et al. (28) reported a significantly lower semen SOD activity in infertile men, as compared with normospermic men although Badade et al. (29) and Khosrowbeygi et al. (30) found lower TAS levels in infertile men compared to fertile men.

Over production of ROS can cause DNA strand breaks, DNA cross-links and chromosomal rearrangements (31). Sperm DNA damage is closely related to male infertility and 8-OHdG is a sensitive marker of oxidative DNA damage (14). Some investigators reported higher levels of DNA damage in infertile subjects than healthy fertile men while Aksoy et al. showed the higher levels of 8-OHdG in ejaculated sperm in infertile men (32, 33). The present study showed that the 8-OHdG levels were significantly higher in infertile men compared (p=0. 02) with fertile men (Table II) that was also in accordance with the findings of Kodama et al. (32).

Various studies showed that lipid peroxidation impacts the sperm concentration, motility, morphology and associated with poor sperm quality (25, 34). Asbagh et al. and Patel *et al*. showed that there was a significant association between semen MDA and abnormal sperm morphology, and decrease semen TAC and weak sperm motility (35, 36). In the present study, the percentage of normal cells was 6.73% (Table I) in infertile men that is slightly higher than the reference value (<4%) set by WHO and that might be because of different population. Here in this study, MDA and PHP were negatively correlated (p<0.001) with sperm parameters while there was a positive correlation (p<0.001) between sperm parameters and SOD and TAS (Fig. 1a to 1d; Fig. 2a to 2d). Murawski et al. showed a positive correlation between SOD activity in seminal plasma and semen quality parameters (28). The inverse correlation between lipid peroxidation and sperm motility has been shown by Keskes-Ammar et al. (37). 

A positive correlation of 8-OHdG with MDA and PHP (p<0.01 and p<0.001, respectively) and an inverse correlation of 8-OHdG with SOD, TAS, motility, sperm count and morphology (in all cases p<0.001) was found (Fig. 4a to 4d; 1e, 2e and 3e), which clearly indicated the effect of oxidative stress on the sperm DNA damage hence their fertility potentials. This observation was in agreement with the Ni et al. study, which indicated a negative correlation between the percentage of sperm with DNA damage and the standard sperm parameters and 8-OHdG level and sperm density and sperm number (38).

## Conclusion

In conclusion, positive correlation of SOD and TAS with sperm parameters and negative correlation with MDA and PHP indicated oxidative stress has a deleterious effect on male infertility. On the other hand, negative correlation of 8-OHdG with sperm parameters, SOD and TAS and positive correlation with MDA and PHP indicated that ROS induced sperm DNA damage might have a significant role in the etiology of impaired sperm functions. Thus, evaluation of seminal MDA, PHP, SOD, TAS and 8-OHdG could be a valuable diagnostic tool for defining sperm fertilization potential. However, additional studies and large scale trials are needed to further elucidate and define the mechanisms of sperm DNA damage and their clinical significance in reproductive outcomes.
